# Development of lntraepithelial Cells in the Porcine Small Intestine

**DOI:** 10.1155/2001/25301

**Published:** 2001

**Authors:** M. A. Vega-López, G. Arenas-Contreras, M. Bailey, S. González-Pozos, C. R. Stokes, M. G. Ortega, R. Mondragón-Flores

**Affiliations:** 1 Center for Research and Advanced Studies (CINVESTAV-IPN) Dept. Experimental Pathology Av. IPN 2508 Mexico 07360, D.F. Mexico; 2 Electronic Microscopy Unit Av. IPN 2508 Mexico 07360, D.F. Mexico; 3 Dept. Genetics and Molecular Biology Av. IPN 2508 Mexico 07360, D.F. Mexico; 4 Dept of Biochemistry. Av. IPN 2508 Mexico 07360, D.F. Mexico; 5 Laboratory of Human Immunology FES-Cuautitlán-UNAM México Mexico; 6 Division of Molecular and Cellular Biology Department of Clinical Veterinary Sciences University of Bristol UK

**Keywords:** IEL, immune development, lymphocyte, mucosal immunology, small intestine, swine

## Abstract

The number, phenotype, localisation and development of intraepithelial lymphocytes (IEL) from duodenum (Du) and ileum (Il) were studied by immunohistochemistry (IHC) and light and electron microscopy in unweaned (0–7 weeks old) and six months-old pigs. Developmental changes at birth showed that 38% of the total lymphocytes in the villi were IEL, mainly of the CD2+CD4-CD8- double negative (DN) phenotype. That proportion rose to over 50% at week 5 after birth, resembling adult proportion, although still with fewer cells than in adult pigs. CD4+ cells appeared relatively early in life although they were confined to the lamina propria (LP) and CD8+ cells were found only in low numbers. In the villi of adult animals, almost half of the total number of lymphocytes were IEL (49% Du, 52% Il). Over half of these IEL (52% Du, 53% Il) showed the CD2+CD4-CD8+ phenotype and were localized at the epithelium's basement membrane. Numerous (43% Du, 42% Il) DN IEL were found grouped at the enterocyte nucleus level and relatively few (5% in Du and Il) granular IEL were found apically in the epithelium. These proportions were homogeneously maintained along the villi's tip, middle and bottom, suggesting that the IEL may have their origin in the LP. Therefore, the IEL compartment in the porcine intestine develops slowly with age and is actually composed by a heterogeneous population of cells (null, DN and CD8+). These results may explain the increased susceptibility of young animals to disease during the lactation period and should be taken into account when functional studies are carried out with IEL. The quantitative results of this paper established a model for studies on the effect of age, diet, normal flora, infection and oral immunization on the IEL of the gut.


*Developmental Immunology*, 2001, Vol. 8(2), pp. 147-158
Reprints available directly from the publisher
Photocopying permitted by license only

© 2001 OPA (Overseas Publishers Association)
N.V. Published by license under
the Harwood Academic Publishers imprint,
part of The Gordon and Breach Publishing Group,
a member of the Taylor and Francis Group

### Development of Intraepithelial Cells in the Porcine Small Intestine

M.A. VEGA-LÓPEZ[a*], G. ARENAS-CONTRERAS[e], M. BAILEY[f], S. GONZÁLEZ-POZOS[b], C.R. STOKES[f], M.G. ORTEGA[c] and R. MONDRAGÓN-FLORES[d]

*[a]Center for Research and Advanced Studies (CINVESTAV-IPN), Dept. Experimental Pathology, [b]Electronic Microscopy Unit, [c]Dept. Genetics and Molecular Biology and [d]Dept of Biochemistry., Av. IPN 2508, Mexico 07360, D.F., MEXICO, [e]Laboratory of Human Immunology, FES-Cuautitlán-UNAM, México and [f]Division of Molecular and Cellular Biology, Department of Clinical Veterinary Sciences, University of Bristol, U.K*

The number, phenotype, localisation and development of intraepithelial lymphocytes (IEL) from duodenum (Du) and ileum (Il) were studied by immunohistochemistry (IHC) and light and electron microscopy in unweaned (0–7 weeks old) and six months-old pigs. Developmental changes at birth showed that 38% of the total lymphocytes in the villi were IEL, mainly of the CD2+CD4-CD8- double negative (DN) phenotype. That proportion rose to over 50% at week 5 after birth, resembling adult proportion, although still with fewer cells than in adult pigs. CD4+ cells appeared relatively early in life although they were confined to the lamina propria (LP) and CD8+ cells were found only in low numbers. In the villi of adult animals, almost half of the total number of lymphocytes were IEL (49% Du, 52% Il). Over half of these IEL (52% Du, 53% Il) showed the CD2+CD4-CD8+ phenotype and were localized at the epithelium's basement membrane. Numerous (43% Du, 42% Il) DN IEL were found grouped at the enterocyte nucleus level and relatively few (5% in Du and Il) granular IEL were found apically in the epithelium. These proportions were homogeneously maintained along the villi's tip, middle and bottom, suggesting that the IEL may have their origin in the LP. Therefore, the IEL compartment in the porcine intestine develops slowly with age and is actually composed by a heterogeneous population of cells (null, DN and CD8+). These results may explain the increased susceptibility of young animals to disease during the lactation period and should be taken into account when functional studies are carried out with IEL. The quantitative results of this paper established a model for studies on the effect of age, diet, normal flora, infection and oral immunization on the IEL of the gut.

*Keywords:* IEL, immune development, lymphocyte, mucosal immunology, small intestine, swine

*Abbreviations:* BM, basement membrane, Du, duodenum, Il, ileum, IHC, immunohistochemistry, IEL, intraepithelial lymphocyte, DN, double negative, LP, lamina propria, LPL, lamina propria lymphocytes, OM, optical microscopy, PLP, paraformaldehyde-lysine-periodate, TEM, transmission electron microscopy

* Senior author for correspondence: Marco A. Vega-López, PhD, Assistant Professor, CINVESTAV-IPN, Dept. Experimental Pathology, Av. IPN 2508, México 07360, D.F., México. Telephone 0052–5–747–3800 Ext'n 5655, Fax: 0052–5–747–9890. e-mail: mavegal@yahoo.com

### INTRODUCTION

Oral immunization has aroused great interest and expectation since this route may represent an alternative for disease control and also to prevent and control autoimmune diseases by inducing tolerance to autologous antigens (Brandtzaeg, 1995). The mucosal immune system has some similarities but also many differences with the systemic immune system (Stokes, 1988; Kiyono, *et al.*, 1992). On the one hand, both systems contain cells which follow similar developmental patterns and effector functions (McGhee and Kiyono, 1993). On the other, differences in cell distribution and immune regulation have also been described (Kiyono, *et al.*, 1992). Unlike the systemic immunological tissues, the mucosal immune system in the gut must continuously deal with an enormous array of foreign material and must be able to accurately discriminate between harmless food antigens and commensal microorganisms and hazardous material, inducing anergy and/or tolerance to the former and a strong local and systemic immune response against the latter (Bienenstock, *et al.*, 1987; Moqbel and MacDonald, 1990; Stokes, *et al.*, 1994). Moreover, the local immune system must maintains an appropriate balance between the organism and bacteria from the normal flora of the gut by mechanisms such as IgA-mediated immune exclusion (Stokes *et al.* 1975; Walker, 1987; Husby, 1988; Brandtzaeg, 1995). These two functions of the local immune system are likely to be carried out through regulatory mechanisms different to those found in the systemic immune system and, perhaps, through subtle but substantial differences in the type and function of the cells found at the mucosal sites.

The gut contains the largest number of immune cells in the organism, approximately 60% of all lymphocytes (Parrot, 1987; Hamad and Klein, 1994; Takahashi and Kiyono, 1999). Many T cells are located in the lamina propria (LPL) and within the epithelium (intraepithelial lymphocytes, IEL) (Moqbel and MacDonald, 1990). Because of their vicinity to the intestinal lumen and their access to food antigens, IEL are strategically located to induce, regulate and perform immune responses (Pabst, 1987; Cerf-Bensussan and Guy-Grand, 1991; McGhee, *et al.*, 1992). Previous studies in the pig have demonstrated that, unlike rodents and humans, the lymphoid components of the intestinal villi are poorly developed at birth (Vega-Lopez, *et al.*, 1993; Pabst and Rothkotter, 1999). The postnatal development of this compartment appears to be driven by exposure to microbial antigens, since it does not occur in germ-free pigs (Pabst and Rothkotter, 1999) and is accelerated by weaning in conventional pigs (Vega-Lopez, *et al.*, 1995). The pig therefore provides an appropriate system to study the development of IEL compartments in response to antigen. Despite this, and despite extensive studies in other species (Guy-Grand, *et al*, 1978; Tagliabue *et al*, 1981; Flexman, 1983, Brandtzaeg, *et al*, 1989; Ebert, 1989; Jarry, *et al*, 1990, Sanchez-Garcia, *et al.*, 1997), relatively few studies of IEL development in pigs have been undertaken (Wilson *et al.* 1986a; 1986b, Whary, *et al.*, 1995; Pabst and Rothkotter, 1999). Therefore, in this paper we determined the number, phenotype, localization and development of IEL in the pig gut.

### RESULTS

### Developmental Changes In Porcine Intestinal Intraepithelial Cells

The number of CD2+ IEL in unweaned animals (birth up to 7 weeks of age) is shown in Figure 1. The number of CD2+ cells was initially low in epithelium and lamina propria (LP), but increased rapidly thereafter in both compartments (Figure 1). The increase was more evident in Du compared with Il at birth and weeks 3 and 5 (P<0.05) (Figure 2). At birth the epithelium contained 38% of the total CD2+ cells in Du and 35% in Il villi, and that percentage increased steadily thereafter up to 52% and 64% respectively, at 7 weeks of age (data not shown). CD2, CD4 and CD8 staining by immunohistochemistry (IHC) in serial

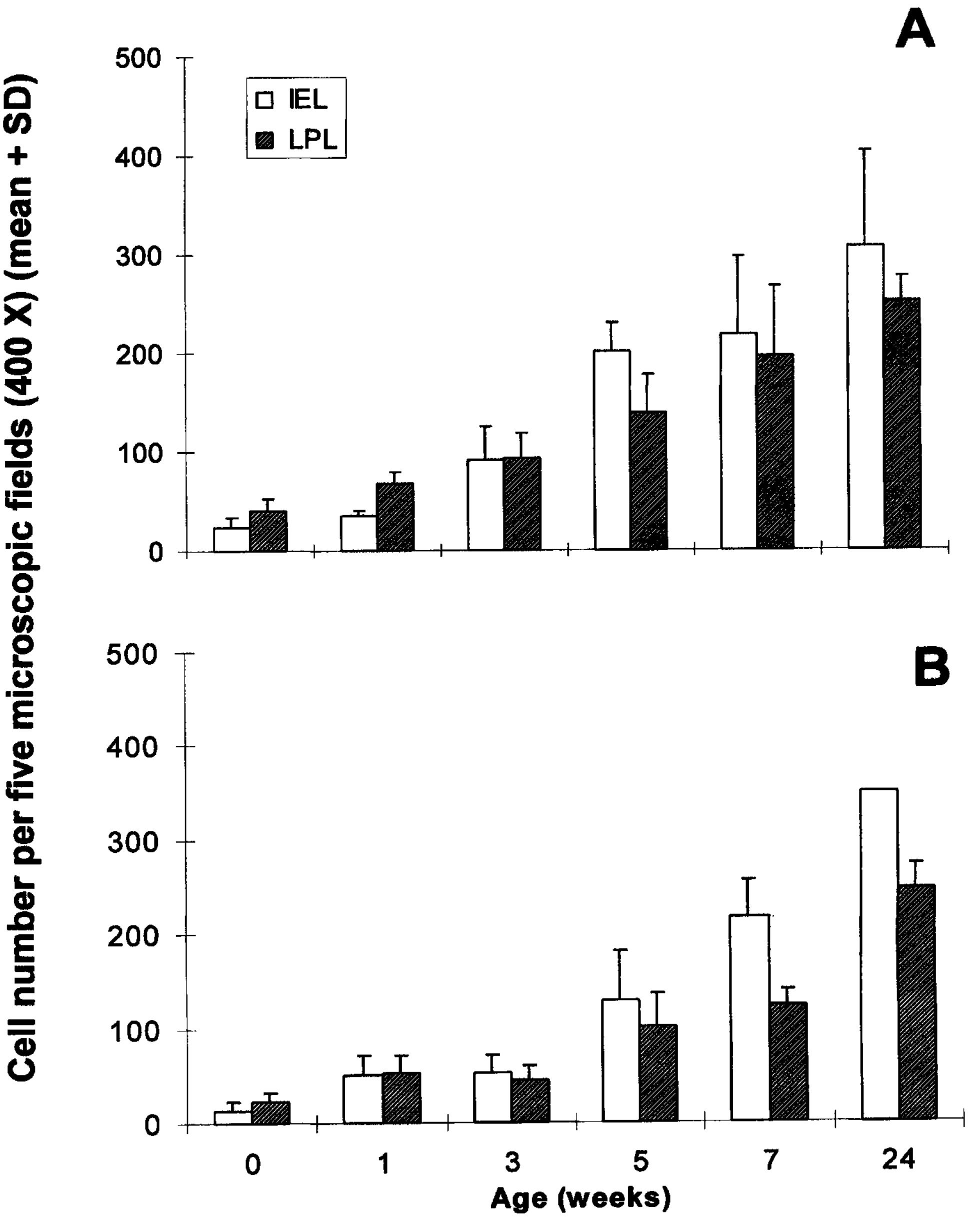

FIGURE 1 Determination of CD2+ cells in duodenum (A) and ileum (B) villi of unweaned (0–7 weeks, n=4 per age group) and conventionally reared adult (24 weeks, n=5) pigs. Each column represents the mean number of intraepithelial (IEL = empty bars) and lamina propria (LPL = slashed bars) CD2+ cells, counted in the villi's epithelium of five randomly chosen microscopic fields (400 X) per animal frozen sections from 4 animals per age group (birth, 1, 3, 5, and 7 weeks) showed that most intestinal cells in young animals were CD2+ double negative (DN) (figure 3). CD4+ cells appeared early in the intestinal LP (figure 3), but not in the epithelium and CD8+ cells appeared late in life and remained low in number (figure 3).

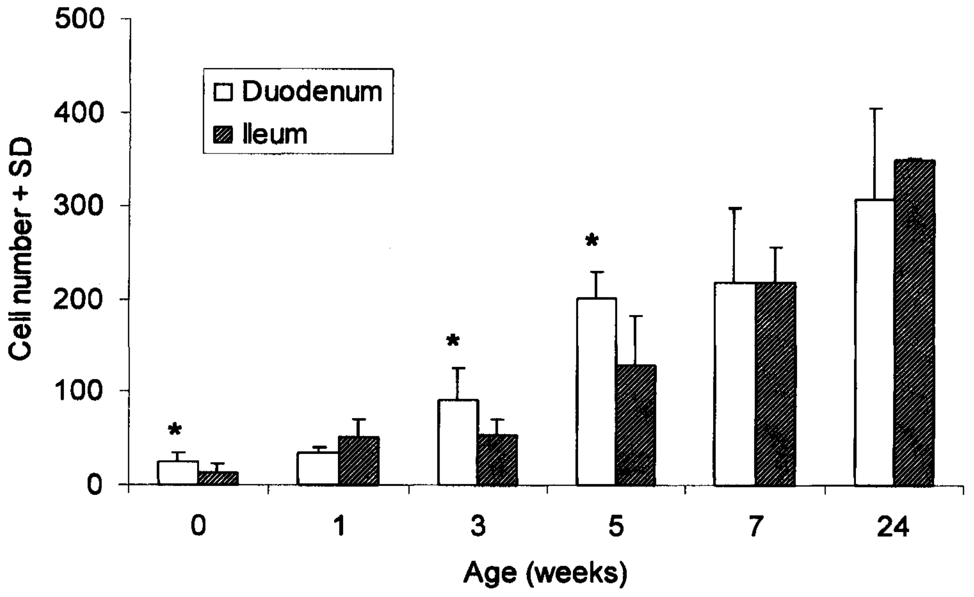

FIGURE 2 Development of intraepithelial CD2+ cells in the small intestine of unweaned (0–7 weeks old, n = 4 animals per age group) and conventionally reared adult animals (24 weeks old, n=5). Each column represents the mean number of IEL of duodenum (empty bars) and ileum (slashed bars), counted in the villi's epithelium of five randomly chosen microscopic fields (400 X) per animal. The asterisks above the bars represent statistical difference (P<0.05) between duodenum and ileum at that time point by paired t-Student test

### Determination of the Number, Phenotype and Topological Distribution of Intraepithelial Cells in the Small Intestinal Villi of Adult Pigs

The average number of total lymphocytes per villus (IEL+LPL) in five adult animals is shown in Table I. Up to half of these cells were located at the epithelium compartment in duodenum (Du) (49%) and ileum (Il) (52%) (Table I). The proportion of intraepithelial cells was homogeneously distributed from the tip (32% Du, 34% Il), through the middle (37% Du, 39% Il), and the bottom (31% Du, 27% Il) of the villi's epithelium (Figure 4A and Table I). The topological distribution of IEL within the epithelium (Figure 4B) showed that half of them located preferentially at the basement membrane (BM) (52% Du, 53% Il) of the tip, middle and bottom of the villi. Another important proportion of cells (43% Du, 42% Il) was found around the enterocyte nucleus and only a small proportion (5% Du, 5% Il) of cells was located at the epithelium's apical zone (Table I). Immunohistochemical staining of serial frozen sections from 6 months old animals (n = 5), showed that most IEL at the BM were CD2+CD4-CD8+ cells (Figure 3). Whereas IEL localized at the enterocyte's apical and nuclear level were mostly DN cells (Figure 3). It was clear that most of the CD4+ cells were confined to the villus LP (Figure 3).

### Ultrastructural Study of Small Intestinal Intraepithelial Cells in Adult Pigs

Cells morphologically similar to lymphocytes and with intraepithelial distribution were detected by transmission electron microscopy (TEM) in duodenal and ileal samples from five six months old pigs. These cells ranged in size from 2.5-to 7 μm of diameter. At the epithelium's apical level (Figure 5A), the IEL were mainly round cells of approximately 5 μm in diameter and with numerous long cytoplasmic prolongations interlaced with the enterocyte's plasma

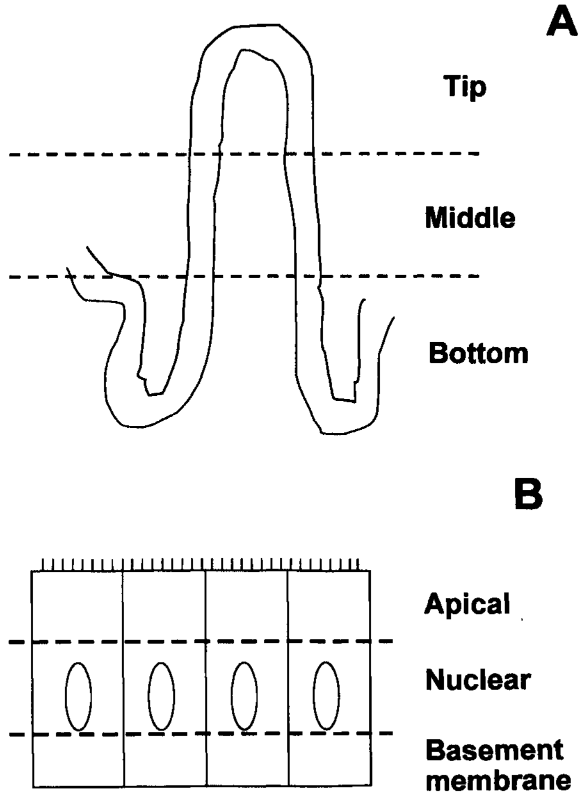

FIGURE 4 Diagram of a small intestinal villus (A) and the epithelium (B) which shows the areas into they were divided to determine the number and localization of intraepithelial cells in six months old pigs membrane (Figure 5B). Dense granules of 0.6 μm in diameter and many ribosomes were detected in the cytoplasm. Besides, adjacent epithelial cells had a high number of small vesicles of 0.2 μm in diameter (Figure 6A). Many IEL were located at the epithelial nuclear zone, some of them were elongated, reaching up to 7 μm of length (Figure 5C). The cytoplasm was twice as large as the nucleus and several mitochondria, ribosomes and endoplasmic reticulum were detected there (Figure 5C). Two or more cells in the same intravacuolar space were frequently seen in this area of the tissue (Figure 3G, Figure 6A). The smallest IEL (2.5 μm in diameter), were detected near the BM (figure 5A). These cells were nearly spherical in shape and located into a vacuole in the intraepithelial space (figure 5D). Their nuclei were prominent with few cytoplasm volume and organelles. Moreover, cells leaving the epithelium were occasionally detected although in low number. These cells did not show morphological features of apoptosis (figure 6B), showing nuclei and cytoplasm integrity.

### DISCUSSION

At the intraepithelial compartment, a large, heterogeneous group of cells has the potential for early contact with antigenic material derived from the gut (Cerf-Bensussan and Guy-Grand, 1991). The ability of normal individuals to discriminate between harmless dietary components and potentially hazardous material and to mount appropriate immune responses implies the presence of reliable but yet not well identified regulatory mechanisms. Previous studies have quantified (Ferguson, 1978, Parrot, 1987) and characterized human and rodent IEL in terms of morphology (Marsh, 1980), phenotype (Brandtzaeg, *et al.*, 1989; Jarry, *et al.*, 1990; Lefrancois, 1991; van Kerckhove, *et al.*, 1992; McGhee and Kiyono, 1993) and effector functions (Ebert, 1989; Fujihashi, *et al.*, 1990; Guy-Grand, *et al*, 1991; McGhee, *et al.*, 1992; Mega, *et al*, 1992; Beate, *et al.*, 1996; Fujihashi, *et al.*, 1997). It has also been reported that cells with heterogeneous characteristics are found at the epithelial compartment (Tagliabue *et al.*, 1981; Flexman, 1983; Lefrancois, 1991; Christ and Blumberg, 1997). Besides, some authors have suggested that the granular cells in the epithelium may be a population of mast cell precursors (Flexman, 1983). However, little information is available for other species including the pig (Chu *et al.*, 1979; Wilson, *et al.*, 1986a, 1986b; Olivier, *et al.*, 1994; Whary, *et al.*, 1995), a species whose similarities with human anatomy and physiology makes it a good experimental model (Tumbleson, 1986). Moreover, little information is available in any of these models about the IEL distribution along the villi and within the epithelium and therefore, quantitative studies on these compartments are needed. In this paper the number, phenotype, localization and development of lymphocytes lodged in these different epithelial compartments of the small intestine are described.

TABLE I The number and localisation of intraepithelial cells in the porcine small intestine. Numbers represent the mean and standard deviation (SD) of cells counted from 10 villi in duodenum and 7 villi in ileum in each of five six-months old healthy pigs. The data was analysed by paired t-Student test. IEL = intraepithelial lymphocytes, LPL = lamina propria lymphocytes

| | *Duodenum* | | *Ileum* | |
|---|---|---|---|---|
| | *Mean (SD)* | *%* | *Mean (SD)* | *%* |
| **Lymphocytes in a villus** | | | | |
| IEL's | 112 (6) | 49 | 99 (16) | 52 |
| LPL | 116 (17) | 51 | 89[a] (13) | 48 |
| **IEL localization along the villus** | | | | |
| Tip | 35 (12) | 32 | 33 (11) | 34 |
| Middle | 42 (13) | 37 | 39 (11) | 39 |
| Bottom | 35 (15) | 31 | 27 (9) | 27 |
| **IEL localization within the epithelium** | | | | |
| Apical | 5 (2) | 5 | 5 (2) | 5 |
| Nuclear | 48 (13) | 43 | 42 (11) | 42 |
| Basement membrane | 59 (17) | 52 | 52 (15) | 53 |

a. P = 0.033 with duodenum

It is well established that young animals are quite susceptible to infection. This may correlate with the very low numbers of LPL and IEL present at birth and with their relatively subsequent slow accumulation (Vega-Lopez *et al.*, 1993; Pabst and Rothkotter, 1999). In this paper it was shown that over 50% of the total T cells in the small intestine were located at the epithelium in 6 months old animals, pointing out the importance of these cells located at the very entrance of foreign material. Therefore it was of interest to study the way those cells reach that proportion in the young pig. Although the total number of intestinal cells was low at birth, a significant proportion (38 and 35% in Du and Il) was in the epithelial compartment. Most of these cells were DN since CD4+ and CD8+ cells are only detectable later in life (Figure 3, Vega-López *et al.*, 1993; Pabst and Rothkotter, 1999). This is an interesting finding considering that the animal comes from a virtually sterile environment, where antigenic stimuli are rather limited, suggesting that this IEL population may have a role distinct to protection. Soon after birth there was a dramatic increase of CD2+ IEL, especially in weeks 3 and 5 in Du where these increases were higher than those found in Il (P<0.05) (Figures 2 and 3). These changes may suggest that the stimuli for IEL, to reach their final localization, might come from the mouth (food antigens), although the normal microflora might also contribute to these antigenic stimuli in driving lymphocytes to the epithelial compartment, rather to the LP, even in the absence of contact with solid food, as it has been demonstrated at weaning (Vega-Lopez, *et al.*, 1995). The absolute number of cells in young animals was still far less than that of adults, a feature that may account for the high susceptibility of the young pig to enteric infections.

At birth, most of the intestinal lymphoid cells were DN (NK?) and CD4+ cells were soon evident, although mainly located at the LP (Figure 3A, B, E). In contrast, CD8+ cells appeared relatively late in life (Figure 3C, F, Vega-Lopez *et al*, 1995). This sequential development and cell localization in the gut might be related to function, and studies in this respect are required to clarify their role in the intestinal immune regulation.

The origin of the IEL is still obscure, but since these cells were uniformly distributed along the tip, middle and bottom of the villi in adult animals (Table I), they may migrate from the LP to the epithelium, where they are selectively retained in the vicin-

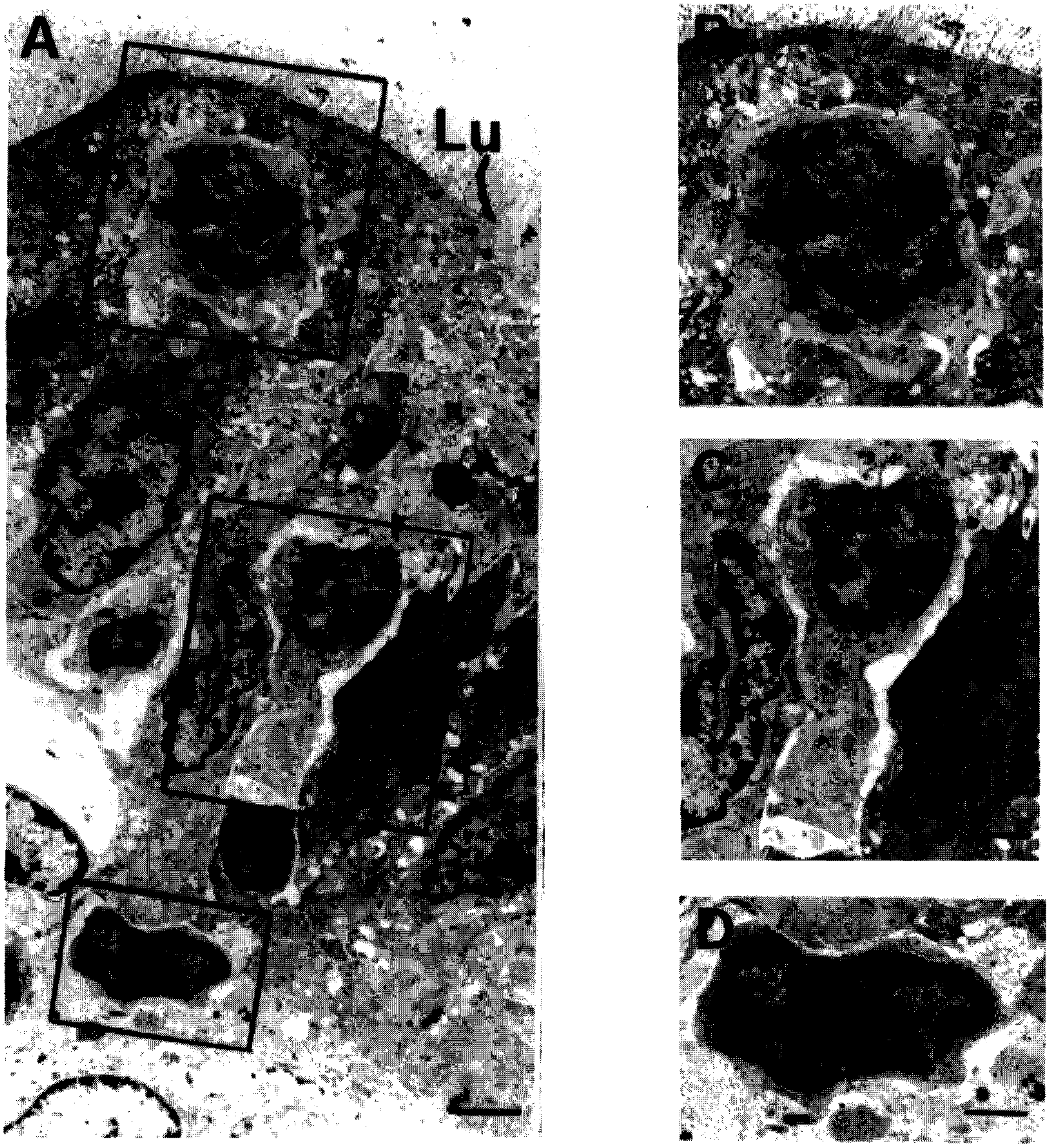

FIGURE 5 Representative transmission electron micrograph of porcine duodenal epithelium (A). Intraepithelial cells can be distinguished at the apical (B), nuclear (C), and basement membrane (D) zones. Sample taken from a six-month old pig. Identical results were found in five different animals in duodenum and ileum. Lu = intestinal lumen, e = enterocyte. Scale bar A = 2 μm, B, C and D = 1 μm ity of the BM (Figure 3), where the highest proportion of IEL was located (52 and 53% in Du and Il, Table I). Most of these cells bear the CD2+CD4-CD8+ phenotype, suggesting a cytotoxic and regulatory role for them. This particular location is similar to that found in other species and may allow these cells to interact with adjacent LP CD4+ (Figure 3I) and MHC-II+ cells (Vega-López *et al.*, 1993).

However, another important proportion of IEL was located at the enterocyte nuclear level (43 and 42% in

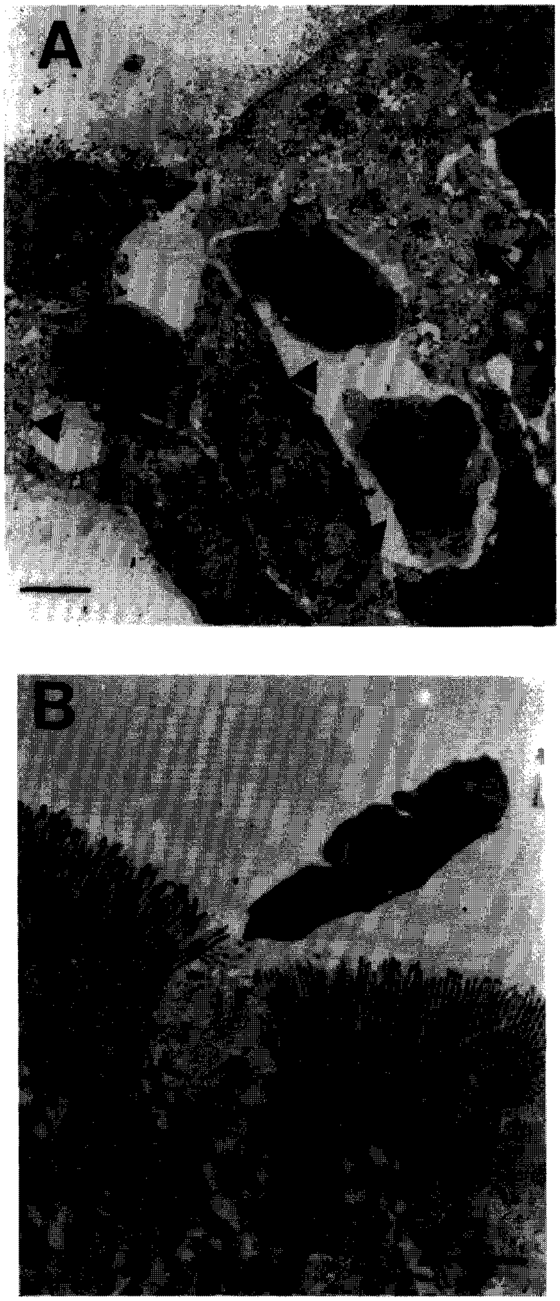

FIGURE 6 Representative transmission electron micrography of porcine duodenal epithelium from a 6-month old animal. Three IELs occupy one intraepithelial space (A, arrowheads). The adjacent enterocyte shows a highly vacuolised cytoplasm (A, arrow). An apparently intact IEL is extruded from the epithelium (B), leaving a disrupted enterocyte behind (B, arrow). Similar results were found in five different animals in duodenum and ileum. Scale bar = 2 μm

Du and Il, Table I) and other less numerous (5% in both Du and Il, Table I) apically near the gut lumen. These cells were mainly DN in phenotype (Figure 3G), as previously described (Vega-Lopez, *et al.*, 1993). The role of these cells is still unknown but they may be composed by an heterogeneous group of cells, including TCR1 (γδ) T cells and NK cells, related to the innate immune response to foreign material as described in humans (Lefrancois *et al.*, 1997) and rodents (Beagley & Husband, 1998).

Apical IELs were mostly granular with prominent cytoplasmic prolongations interlaced with the enterocyte's plasma membrane that showed loss of microvilli and a highly vacuolated cytoplasm (Figures 5A, B and 6A). Some authors have suggested that the apically located IEL is an apoptotic population of cells in the process of extrusion towards the intestinal lumen (Viney & McDonald, 1990). However, TEM ultrastructural analysis of such cells showed no evidence of cell shrinkage or nuclear chromatin condensation and fragmentation (figure 5B), characteristic features of apoptotic cells. These features were also absent in lymphocytes shed into the intestinal lumen (figure 6B), suggesting possible differences between pigs and mice, and stressing the possibility of these cells being extruded by other causes such enterocyte destruction. Considering the low proportion of cells found at the apical zone, the shedding of cells may not be a frequent event in normal (uninfected) animals, but may be related to the elimination of damaged or infected enterocytes. This situation may be different in infected animals. Immune localisation of apoptotic molecules may further clarify this point.

At the enterocyte's nuclear level, there were also IEL with long cytoplasmic processes suggesting active motion. In many cases they were found grouped, suggesting a pattern of traveling through the epithelium following each other (Figures 3G, 5A and 6A). If this was the case, these cells never accumulated at the apical zone of the enterocyte, perhaps because the antigenic stimuli are given at the basolateral part of the enterocyte (Christ and Blumberg, 1997). The heterogeneous morphology and phenotype of the IEL showed that they may be in different stages of differentiation/activation or that they may have different origins (mast cell precursors?), further phenotype identification is needed to clarify their nature.

Finally, it is clear that the epithelial compartment of the gut contains a numerous and rather heterogeneous population of immune cells. This heterogeneity may explain some of the different responses the intestinal immune system is able to produce either inducing tolerance/anergy to harmless diet components or eliciting strong immune responses to hazardous material. This heterogeneity must be taken in consideration when isolation protocols and *in vitro* functional studies are performed. These quantitative studies established an experimental model to evaluate, in a more precise manner, the effects of antigen stimuli (diet, normal flora, infection, oral immunization, etc.) on the cells populating the epithelial compartment and may allow the study of the relationships among cell localisation, phenotype and function of IELs.

### MATERIAL AND METHODS

### Pigs

To determine the number, distribution and ultrastructural studies of cells within the small intestine, five 6 months old healthy pigs, from a minimal disease and enzootic pneumonia-free herd were used. To study the changes in IEL populations with age, at least four unweaned littermates per age group (birth, 1, 3, 5 and 7 weeks old) were used. The animals were maintained on the sow without access to solid food until slaughtered. The animals were housed in the animal facility of the Department of Clinical Veterinary Sciences, University of Bristol, UK.

### Processing of Samples for Optical (OM) and Transmission Electron Microscopy (TEM) Studies

Samples of Du and Il from each animal were taken immediately after the animals were sacrificed with an intravenous overdose of sodium pentobarbitone (Euthatal, RMB Animal Health, Ltd., Dagenham, UK). The samples from the young pigs, were washed in cold PBS and fixed in paraformaldehyde-lysine-periodate (PLP) as previously described (Vega-Lopez *et al.*, 1993). For ultrastructural studies, intestinal samples from adult animals, previously immersed in cold PBS, were fixed in 2.5% glutaraldehyde (Electron Microscopy Sciences, Forth Washington, PA) in PBS for 60 minutes at room temperature. After washing in PBS, the tissues were post-fixed with 1% osmium tetroxide in PBS for 60 minutes at 4 C. Following rinsing and dehydration with increasing concentrations of ethanol, the specimens were embedded in Spurr's resin (Embedding kit, Polysciences, Inc.) following the manufacturer's instructions. Sections of 0.5 μm for OM and 100 nm for TEM were then cut on an ultramicrotome (Ultracute, Reichter-Jung) and finally stained with 2% uranile acetate and 0.4% of lead citrate for observation and micrography with a JEM-2000EX electron microscope (JEOL Ltd, Tokyo, Japan) on Kodak Electron Image Film (Eastman Kodak, Co., Rochester, NY).

### Analysis of Intestinal Tissue Samples

IHC stained cells were counted in LP and epithelium of at least five randomly chosen microscopic fields (400 X) from each intestinal pig sample. The area of the tissue was measured using a semiautomatic, computer assisted image analyser (VIDS V, Synoptics, Ltd., UK) and positive cells within that area were counted and recorded. The number and localisation of intraepithelial cells were analysed in intestinal samples of adult pigs stained with Harris haematoxylin and the cells with lymphocyte morphology were counted in at least 10 villi from Du and 7 villi from Il from each one of five animals, avoiding the Peyer's patches villi. The villi were randomly selected on the basis of similar length and angle of cut. Each villus was divided in three proportionally equal parts: tip, middle and bottom (Figure 5A), from the villus tip to the deepest part of the crypts. Intraepithelial cells from each of these parts were counted and recorded. In the cell localization within the epithelium study, the epithelium was divided in three zones: apical, nuclear and basement membrane (Figure 5B). The cells located in these compartments in the villi of each sample were counted and recorded using the image analyser. For ultrastructural cell characteristics and detailed localisation of IEL, Du and Il TEM photographs were taken from five adult animals and characteristic morphological and topological features were analysed and recorded.

### Immunohistochemistry

Anti-CD2 (MSA-4, Hammerberg and Schurig, 1986), anti-CD4 (74–12–4, Pescovitz *et al.*, 1984) and anti-CD8 (76–2–11, Pescovitz *et al.*, 1984) monoclonal antibodies (MAb) were used in IHC. For the developmental study, MSA-4 positive cells in the epithelium and LP of the Du and Il villi, from at least five randomly chosen fields (400 × magnification) were counted and recorded from paraffin embedded samples from at least four young unweaned pigs (0, 1, 3, 5 and 7 weeks old) in each age group. Du and Il frozen samples from five 6 months old animals were stained with MSA-4, 74–12–4 and 76–2–11 MAb by IHC, to determine the phenotype and localization of IEL within the epithelium by OM. The cells were counted and recorded using the image analyser. Paired t-Student test was used to compare data between age groups and between intestinal sites (Du vs Il).

### *Acknowledgements*

This work was partially funded by CONACyT-México (Projects 4263-M and 26361-B) and the BBSRC of the United Kingdom. GAC received partial support from "Cátedra de investigación (1.24)" from FES-Cuautitlán-UNAM-México. The authors wish to thank BSc Mónica Mondragón for technical assistantship and Professor Derek Wakelin (U. Nottingham, UK) for critically reading the manuscript of this work.

### *References*

Beagley K.W. and Husband A.J. (1998). Intraepithelial lymphocytes: Origins, distribution and function. Crit. Rev. Immunol. **18**:237–254.

Beate C., Sydora B.D., Jamieson R. A. and Kronenberg M. (1996). Intestinal Intraepithelial Lymphocytes Respond to Systemic Lymphocytic Choriomeningitis Virus Infection. Cellular Immunology **167**:161–169.

Bienenstock J., Ernst P.B. and Underdown B.J. (1987). The gastrointestinal tract as an immunologic organ: State of the art. Annals of Allergy **59** (part II):17–20.

Brandtzaeg P., Halsrensen T.S., Kett K., Krajci P., Kvale D., Rognum T.O., Scott H. and Sollid L.M. (1989). Immunology and immunopathology of human gut mucosa: Humoral immunity and intraepithelial lymphocytes. Gastroenterology **97**:1562–1584.

Brandtzaeg P. (1995). Mucosal Immunology. A long way to the surface. The Immunologist **3**(3):75–77.

Cerf-Bensussan N. and Guy-Grand D. (1991). Intestinal intraepithelial lymphocytes. Gastroenterol. Clin. N. Am. **20**:549–576.

Christ A.D. and Blumberg R.S. (1997). The intestinal epithelial cell: Immunological aspects. Springer Semin. Immunopathol. **18**:449–461.

Chu R.M., Glock R. and Ross D. (1979). Gut associated lymphoid tissues of young swine with emphasis on dome epithelium of aggregated lymph nodules (Peyer's patches) of the small intestine. Am. J. Vet. Res. **40**:1720–1728.

Ebert E.C. (1989). Proliferative responses of human intraepithelial lymphocytes to various T-cell stimuli. Gastroenterology **97**:1972–1381.

Ferguson A. (1978). Lymphocytes and cell mediated immunity in the small intestine. Adv. Med. **14**:278–293.

Flexman J.P. (1983). Natural cytotoxicity responsiveness to interferon and morphology of intraepithelial lymphocytes from the small intestine of the rat. Immunology **48**:733–741.

Fujihashi K., Taguchi T., McGhee J.R., Eldrige J.H., Bruce M.G, Green D.R., Singh B. and Kiyono H. (1990). Regulatory function for murine intraepithelial lymphocyte T cells abrogate oral tolerance. J. Immunol. **145**(7):2010–2019.

Fujihashi K., Kweon M-N., Kiyono H., VanCott J.L., van Ginkel F.W., Yamamoto M. and McGhee J.R. (1997). A T cell/B cell/epithelial cell internet for mucosal inflammation and immunity. Springer Semin. Immunopathol. **18**:477–494.

Guy-Grand D., Griscelli C. and Vassalli P. (1978) The mouse gut T lymphocyte, a novel type of T cell: nature, origin, and traffic in mice in normal and graft-versus-host conditions. J. Exp. Med. **148**:1661–1677.

Guy-Grand D., Cerf-Bensussan N., Malissen B., Malassis-Seris M., Briottet C. and Vassalli P. (1991). Two gut intraepithelial CD8+ lymphocyte populations with different T cell receptors: a role for the gut epithelium in T cell differentiation. J. Exp. Med. **173**:471–481.

Hamad H. and Klein J.R. (1994). Phenotypic and functional heterogeneity of murine intestinal intraepithelial lymphocytes defined by cell density: Implications for route of differentiation and responsiveness to proliferation induction. Immunology **82**:611–616.

Hammerberg C. and Schurig G. (1986). Characterization of monoclonal antibodies directed against swine leukocytes. Vet. Immunol. Immunopathol. **11**:107–121.

Husby S. (1988). Dietary antigens: Uptake and humoral immunity in man. APMIS **96** (Suppl. 1):5–40.

Jarry A., Cerf-Bensussan N., Brousse N., Selz F. and Guy-Grand D. (1990). Subsets of CD3+ (T cell receptor alpha/beta or gamma/delta) and CD3- lymphocytes isolated from normal human gut epithelium display phenotypical features different from their counterparts in peripheral blood. Eur. J. Immunol. **20**:1097–1103.

Kiyono H., Bienenstock J., McGhee J.R. and Ernst P.B. (1992). The mucosal immune system: Features of inductive and effector sites to consider in mucosal immunisation and vaccine development. Reg. Immunol. **4**:54–62.

Lefrancois L. (1991). Phenotypic complexity of intraepithelial lymphocytes of the small intestine, J Immunol. **147**:1746–1751.

Lefrancois L., Fuller B., Huleatt J.W., Olson S. and Puddington L. (1997). On the front lines: Intraepithelial lymphocytes as primary effectors of intestinal immunity. Springer Semin. Immunopathol. **18**:463–475.

Marsh M.N. (1980). Studies of intestinal lymphoid tissue. III. Quantitative analyses of epithelial lymphocytes in the small intestine of human control subjects and of patients with celiac sprue. Gastroenterology **79**:481–492.

McGhee J.R., Mestecky J., Dertzbaugh M.T., Eldrige J.H., Hirasawa M. and Kiyono H. (1992). The mucosal immune system: From fundamental concepts to vaccine development. Vaccine **10**:75–88.

McGhee J.R. and Kiyono H. (1993). New perspectives in vaccine development: Mucosal immunity to infections. Infectious Agents and Disease **2**:55–73.

Mega J., McGhee J.R. and Kiyono H. (1992). Cytokine and Ig producing cells in mucosal effector tissues: analysis of IL-5 and IFN-gamma producing T cells, T cell receptor expression and IgA plasma cells from mouse salivary gland-associated tissues. J Immunol. **148**:2030–2039.

Moqbel R. and MacDonald A.J. (1990). Immunological and inflammatory responses in the small intestine associated with helminthic infections. In: Parasites: Immunity and pathology. J.M. Behnke, Ed. (Taylor and Francis, UK.), pp249–281.

Olivier M., Berthon P. and Salmon H. (1994). Immunohistochemical localization in the intestine of swine of the cellular and humoral components of the immune response. Vet-Res. **25**(I): 57–65.

Pabst R. (1987). The anatomical basis for the immune function of the gut. Anat. Embriol. Beri. **176**(2):135–144.

Pabst R. and Rothkotter H.J. (1999). Postnatal development of lymphocyte subsets in different compartments of the small intestine of piglets. Vet. Immunol. Immunopathol. **72**:167–173.

Parrot D.M.V. (1987). The structure and organisation of lymphoid tissue in the gut. In: Food allergy and intolerance. J. Brostoff and S.J. Challacombe Eds. (Bailliere Tindall, W.B. Saunders, UK), pp 3–6.

Pescovitz M.D., Lunney J.K. and Sachs D.H. (1984). Preparation and charaacterization of monoclonal antibodies reactive with porcine PBL. J. Immunol. **133**:368–375.

Sanchez-Garcia F.J., Aller W.W. and McCormack W.T. (1997). Impaired calcium mobilization and differential tyrosine phosphorylation in intestinal intraepithelial lymphocytes. Immunology **91**:81–87.

Stokes C.R.. Soothill J.F. and Turner M.W. (1975). Immune exclusion is a function of IgA. Nature (London) **255**:745–746.

Stokes C.R. (1988). Immune systems in the porcine gut. The pig veterinary society proceedings **20**:19–30.

Stokes C.R.. Bailey M. and Wilson A.D. (1994). Immunology of the porcine gastrointestinal tract. Vet. Immunol. Immunopathol. **43**:143–150.

Tagliabue A., Luini W., Soldateschi D. and Boraschi D. (1981). Natural killer activity of gut mucosal lymphoid cells in mice. Eur. J. Immunol. **11**:919–922.

Tumbleson M.E. (1986). Preface. In Swine in biomedical research. Proceedings of a Conference on swine in biomedical research, June 17–20, 1985, University of Missouri, USA (New York:Plenum Press), pg v.

Van Kerckhove C., Russell G.J., Deusch K., Reich K. Bhan A.K., DerSimonian H. and Brenner, M.B. (1992). Oligoclonality of human intestinal intraepithelial T cells. J. Exp. Med. **175**:57–63.

Vega-López M.A., Telemo E., Bailey M., Stevens K. and Stokes C.R. (1993). Immune cell distribution in the small intestine of the pig: Immunohistological evidence for an organised compartmentalisation in the lamina propria. Vet. Immunol. Immunopathol. **37**:49–60.

Vega-López M.A., Bailey M., Telemo E. and Stokes C.R. (1995). Effect of early weaning on the development of immune cells in the pig small intestine. Vet. Immunol. Immunopathol. **44**:319–327.

Vinney J. and McDonald T.T. (1990). Selective death of T-cell receptor $\gamma/\delta+$ intraepithelial lymphocytes by apoptosis. Eur. J. Immunol. **20**(12):2809–2912.

Walker W.A. (1987). Pathophysiology of intestinal uptake and absorption of antigens in food allergy. Annals of allergy **59**:7–16.

Whary M.T.. Zarkower A., Confer F.L. and Ferguson F.G. (1995). Age-related differences in subset composition and activation responses of intestinal intraepithelial and mesenteric lymph-node lymphocytes from neonatal swine. Cellular Immunology **163**: 215–221.

Wilson A.D.. Stokes C.R. and Bourne F.J. (1986a). Responses of intraepithelial lymphocytes to T-cell mitogens: a comparison between murine and porcine responses. Immunology **58**:621–625.

Wilson A.D.. Stokes C.R. and Bourne F.J. (1986b). Morphology and functional characteristics of isolated porcine intraepithelial lymphocytes. Immunology **59**:109–113.

